# Accurate Measurement of Blast Shock Wave Pressure by Enhanced Sensor System Based on Neural Network

**DOI:** 10.3390/s25196187

**Published:** 2025-10-06

**Authors:** Fan Yang, Hongzhen Zhu, Deren Kong, Chuanrong Zhao

**Affiliations:** 1 School of Automation, Wuxi University, Wuxi 214105, China; 2 Department of Basic Courses, Wuxi University, Wuxi 214105, China; hzzhu@cwxu.edu.cn; 3 School of Mechanical Engineering, Nanjing University of Science and Technology, Nanjing 210094, China; derenkong@hotmail.com; 4 School of Electrical and Information Engineering, Anhui University of Technology, Ma’anshan 243002, China; 5 Anhui Provincial Key Laboratory of Power Electronics and Motion Control, Anhui University of Technology, Ma’anshan 243002, China

**Keywords:** blast shock wave, sensor system, modeling, compensation, neural network

## Abstract

During blast shock wave pressure measurement, strong mechanical vibrations and shocks can affect the dynamic characteristics of shock wave pressure sensors, introducing measurement errors. To improve measurement accuracy for the compression phase, a specialized buffer device was designed to enhance the sensor’s dynamic response to transient pressure rises. Using a double-diaphragm shock tube, the dynamic calibration of the enhanced sensor system was carried out and the influence of the buffer device on the dynamic performance was investigated. A mathematical model based on a backpropagation (BP) neural network was developed to characterize the sensor system, and a dynamic compensation method was implemented to improve the enhanced shock wave pressure sensor system. Experimental results demonstrated that while the buffer device significantly reduced the operational bandwidth of the sensor system, the BP neural network-based dynamic compensation effectively widened the bandwidth and improved measurement accuracy. This research provides a practical solution for high-precision dynamic pressure measurement, specifically targeting the compression phase in complex environments.

## 1. Introduction

Blast shock wave pressure measurement is critical for assessing the destructive power of explosives. However, the complex blast environment, combined with the steep rising edge and short duration of blast shock wave pressure signals, makes high-accuracy measurement challenging yet highly significant [1,2]. Currently, electrical measurement is the primary method used in explosion tests, with piezoelectric sensors being the preferred choice due to their high overload resistance and wide operating bandwidths [3]. In explosion tests, integrated circuit piezoelectric (ICP) sensors with built-in charge amplifiers are typically employed [4].

During explosion tests, parasitic effects, such as thermal shocks, mechanical shocks, and vibrations [5,6], introduce unwanted noise into the measured signals. To mitigate these effects, the blast shock wave pressure sensor component, which consists of the pressure sensor, the mechanical buffer structure, and silicone grease, is mounted on a test plate for pressure measurement [6]. However, the altered equivalent mass and stiffness of the assembled structure modify the sensitivity and dynamic characteristics of the sensor system compared to its standalone state. Therefore, the sensor system must be calibrated and modeled to determine whether it meets the requirements for undistorted measurements. If not, dynamic compensation is necessary.

Dynamic calibration devices include periodic pressure generators, drop-weight devices, quasi-δ function pressure generators, and shock tubes [7,8,9]. For the dynamic calibration of blast shock wave pressure sensor systems, shock tubes are the most suitable option. They generate a near-ideal step signal with a bandwidth wide enough to excite the resonant frequencies of the pressure sensor system [10,11].

Dynamic calibration data enables the establishment of a sensor system’s dynamic model. As the pressure sensor’s mathematical model underpins dynamic compensation, its modeling method requires careful study. Traditional modeling algorithms mainly include the least squares method and maximum likelihood estimation—these directly generate the system’s transfer function (an intuitive, universal representation) but tend to converge to local minima and rely on the linear time-invariant (LTI) system assumption, thus failing to address the sensor system’s inherent nonlinearities [12,13,14,15].

Dynamic compensation can be carried out via hardware or software. Hardware methods are limited by material and technical constraints, while software methods show better development potential due to their flexibility and reliability. Software-based dynamic compensation mainly includes two approaches: time-domain dynamic error correction and frequency-domain dynamic characteristic compensation. Time-domain correction uses computational methods on output signals, such as frequency-domain correction, superposition integration, and numerical differentiation. However, frequency-domain correction and deconvolution are prone to spectral aliasing, leakage, and picket fence effects, and numerical differentiation integration may cause error accumulation. Therefore, these methods are gradually being replaced by frequency-domain dynamic characteristic compensation [16,17,18].

The latter approach enhances sensor dynamic performance by designing a compensation model connected in series with the sensor, with system modeling and compensation achievable through both traditional algorithms and intelligent optimization techniques [19]. In recent years, academic and industrial studies have actively explored more advanced modeling and compensation methods to address the increasingly complex requirements of dynamic measurement scenarios, with representative approaches including Convolutional Neural Networks (CNNs), Recurrent Neural Networks (RNNs), Gated Recurrent Units (GRUs), and various hybrid optimization algorithms [20,21,22]. CNNs are designed for spatial grid data and excel at capturing spatial hierarchical features, mainly used to process images and grid data. They can efficiently extract information such as the shape and texture of objects. RNNs and GRUs, as typical deep learning models specialized in time-series data processing, demonstrate distinct advantages in capturing temporal dependencies within dynamic sensor signals. This enables them to better adapt to the time-varying nature of sensor outputs, especially in scenarios where the sensor’s dynamic response changes with external conditions. However, these neural network-based methods often come with notable drawbacks: they generally require more complex training processes and depend on larger volumes of high-quality labeled datasets to ensure modeling accuracy. Unfortunately, in blast shock wave measurement, where experimental conditions are often limited by factors like high test costs, strict safety requirements, and the difficulty of replicating explosive environments, it is often challenging to collect such large-scale datasets, which restricts the practical application of RNNs and GRUs in this field.

The neural network algorithm that appeared at the end of the 20th century is one of the most representative intelligent algorithms; therefore, BP neural networks have been widely researched and applied in recent years. A BP neural network minimizes prediction errors through the backpropagation algorithm and is primarily used to fit simple nonlinear mappings. The BP neural network is commonly considered to be the most mature neural network algorithm suitable for modeling [23,24,25]. The BP neural network-based method proposed in this study offers distinct novelties. First, it achieves a good balance between modeling accuracy and computational efficiency, requiring relatively smaller datasets for training while ensuring fast convergence, which makes it well-suited for the constraints of blast test environments. Second, by combining a specialized mechanical buffer device with BP neural network compensation, it addresses both hardware-induced interference, such as mechanical vibrations and thermal shocks, and software-related dynamic characteristic deviations, forming a comprehensive solution. Third, the proposed method is validated through practical explosion tests with 1 kg TNT charges, demonstrating its strong applicability to real blast shock wave measurement.

This paper is organized as follows: Section 2 investigates the blast shock wave pressure sensor component, establishes an enhanced blast shock wave pressure sensor system that is critical for measuring the compression phase of shock waves, conducts dynamic calibration tests using a double-diaphragm shock tube, and obtains the system’s step responses and dynamic characteristics. Section 3 presents the modeling and compensation methodology based on a BP neural network. In Section 4, we develop the pressure sensor system model and design a dynamic compensation model using the BP neural network. The effectiveness of the compensation method is verified through experimental validation, including its application to measuring blast wave pressure signals from explosion tests. This research significantly contributes to improving both the dynamic performance of pressure sensors and measurement accuracy in blast wave studies that rely on precise compression-phase data for analysis.

## 2. Dynamic Calibration of Enhanced Blast Shock Wave Pressure Sensor System

### 2.1. Blast Shock Wave Pressure Sensor Component

**Figure 1:**
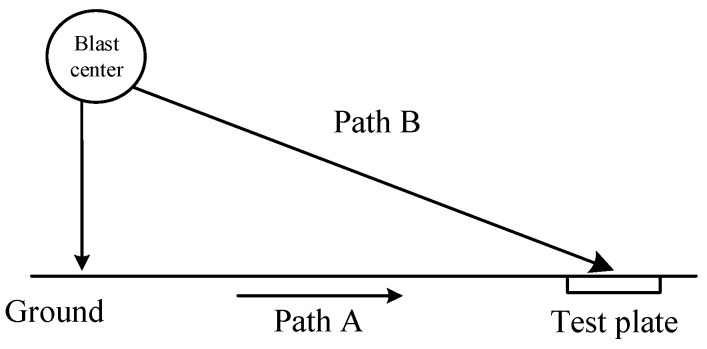
Diagram of shock wave propagation path.

During an explosion, the rapid expansion of explosive products in the air compresses the surrounding atmosphere and generates a blast shock wave. The compression phase of the blast shock wave, characterized by transient high pressure and a steep pressure rise front, is the core dynamic feature that drives subsequent interactions [26]. When a blast shock wave propagates and impacts the ground surface, it generates reflections, which can be classified into three types—normal reflection, regular oblique reflection, and irregular oblique reflection—with the propagation paths illustrated in Figure 1.

After the wavefront of a vertically incident shock wave comes into contact with the ground surface, most of its energy propagates vertically backward in the form of a reflected shock wave, while a small portion is transmitted through the ground as seismic waves that propagate horizontally, as shown by Path A in Figure 1. When the shock wave undergoes regular oblique reflection, it is similarly divided into a reflected shock wave propagating vertically backward and seismic waves propagating horizontally. Due to the separation between the reflected wavefront and the incident wavefront, a specific point on the ground within the regular reflection zone will be impacted twice—once by the incident shock wave and once by the reflected shock wave. When the incident angle exceeds the critical angle, irregular reflection occurs. At this point, part of the shock wave’s energy is transmitted through the ground, causing significant vibration and deformation. The other part of the energy is converted at the air–ground interface into explosive seismic waves that propagate outward through the air, as shown by Path B in Figure 1.

The mechanical shock and vibration in explosive field parasitic effects primarily originate from three sources: vibrations induced by seismic waves generated when the shock wave interacts with the ground; ground vibrations caused by the shock wave’s impact, which subsequently excite vibrations in objects placed on the surface; and direct shock wave interaction with the test plate equipped with the piezoelectric pressure sensor, leading to interference in the sensor’s output. Additionally, the high-temperature thermal shock effect influences shock wave pressure sensors through the following mechanisms: the explosion generates intense heat, causing thermal drift in the sensor’s piezoelectric crystal, while the sensor undergoes thermal stress, resulting in an extraneous thermal response in its output signal.

**Figure 2:**
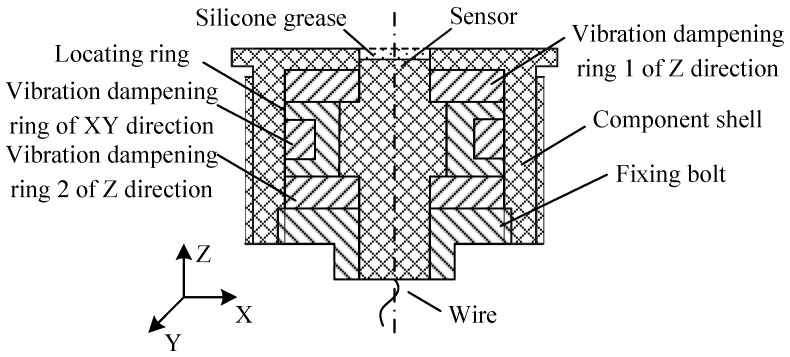
Structure of the sensor component with the buffer device.

To suppress these disturbances, a specialized buffer device [27] was designed to improve measurement accuracy, consisting of structural steel housing and three orthogonally arranged rubber damping rings (density: 0.92 g/cm^3^; tensile strength: 20 MPa; Poisson’s ratio: 0.35), including one vibration dampening ring in the XY direction (6.3 mm inner diameter, 8.3 mm outer diameter) and two vibration damping rings in the Z direction (3.0 mm thickness), all assembled via interference fitting, along with a structural steel fixing bolt and locating ring. The sensor’s sensitive surface was coated with 0.3 mm silicone grease for thermal insulation (Figure 2). This additional structure and insulation layer modify the system’s dynamic characteristics, necessitating dynamic calibration experiments to establish an accurate system model and implement proper dynamic compensation for the sensor system.

### 2.2. Dynamic Calibration of Enhanced Sensor System Based on Double-Diaphragm Shock Tube

A double-diaphragm shock tube consists of three chambers, high-pressure, medium-pressure and low-pressure chambers, and generates a near-ideal step signal [15] at the low-pressure chamber outlet during dynamic calibration tests. Characterized by a 6 ms platform duration and sub-1 μs rise time, this step signal provides an exceptional 1 kHz–1 MHz calibration bandwidth that fully encompasses the pressure sensor system’s operational range, making the system perfectly suited for shock wave pressure sensor system calibration. Compared to single-diaphragm designs, the double-diaphragm configuration offers precise rupture timing control through regulated gas release from the medium-pressure chamber while simultaneously delivering superior performance with higher peak pressures and significantly reduced rise times.

**Figure 3:**
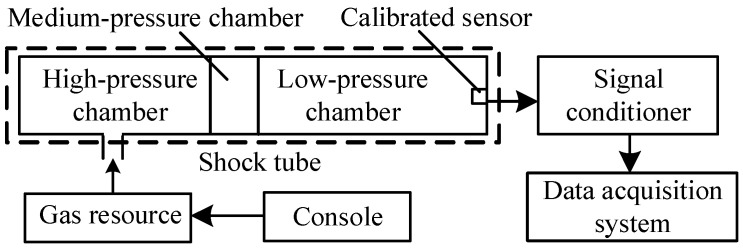
The composition of the dynamic calibration system.

The dynamic calibration system comprises five key components: a gas source, a shock tube, an enhanced pressure sensor component, a signal conditioner, and a data acquisition system, as shown in Figure 3. The traceability process for the step response pressure amplitude is relatively complex, making it difficult to obtain an accurate response signal amplitude. However, the purpose of dynamic calibration is to study the frequency characteristics of the sensor system, which are independent of the amplitude of the response signal. Therefore, there is no need to focus on the amplitude of the step response signal. The normalized dynamic characteristics of the sensor system can be studied by normalizing the step response signal. Consequently, this test configuration eliminates the need for a standard reference sensor. The experimental setup positions only the calibrated sensor component at the precise center of the shock tube’s end wall, connected in series with a signal conditioner, enabling the data acquisition system to capture the step response waveform for subsequent analysis and processing.

**Figure 4:**
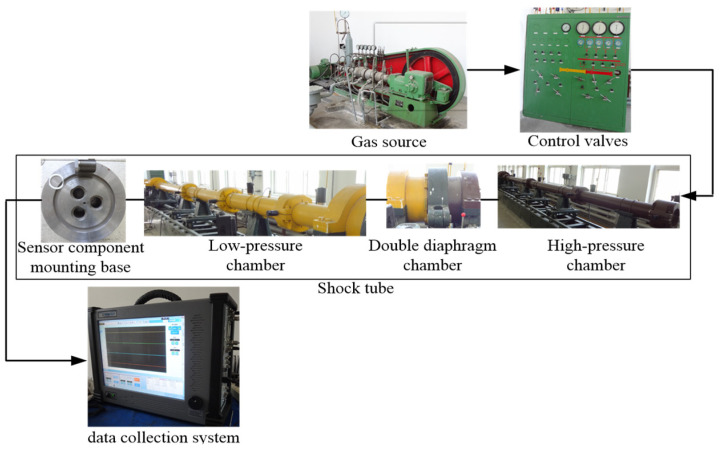
The composition of the dynamic calibration system.

The dynamic calibration experiment for the shock wave pressure sensor system was conducted using a 25 m long shock tube with an inner diameter of 0.25 m, equipped with a 9 m high-pressure chamber. Air was used as both the driver gas and driven gas, and no standard reference sensor was employed. The ICP-type PCB113B sensor with built-in signal conditioning was chosen to build the enhanced sensor component. The PCB113B sensor is a piezoelectric pressure sensor, featuring a fast dynamic response, a wide operating frequency bandwidth, a high measurement accuracy, excellent resistance to harsh environments, and easily processable output signals. It is suitable for shock wave pressure measurement in harsh environments and for shock wave pressure signals with wide effective frequency bandwidths. The dynamic calibration system is illustrated in Figure 4.

### 2.3. Impact Analysis of the Buffer Device on Dynamic Characteristics

To assess the dynamic characteristic variations induced by mechanical enhancements and thermal insulation, dynamic calibration experiments were performed on three progressively modified sensor configurations: the baseline PCB113B sensor mounted directly on the base, the sensor integrated into the mechanical buffer structure, and the complete assembly incorporating both the buffer structure and a 0.3 mm silicone grease layer applied to the sensitive surface, all properly mounted on the end of the shock tube.

The dynamic characteristics of the pressure sensor system can be obtained based on the differential method. The differential method is used to obtain dynamic characteristics by building a differentiator. First, we derived the normalized step response y(t). Then, a digital differentiator was designed to differentiate it. Moreover, the impulse response h(t) can be obtained by h(t) = dy(t)/dt. And finally, by Fourier transform, the dynamic characteristics H(jω) of the pressure sensor can be calculated by H(jω) = F[h(t)].

**Figure 5:**
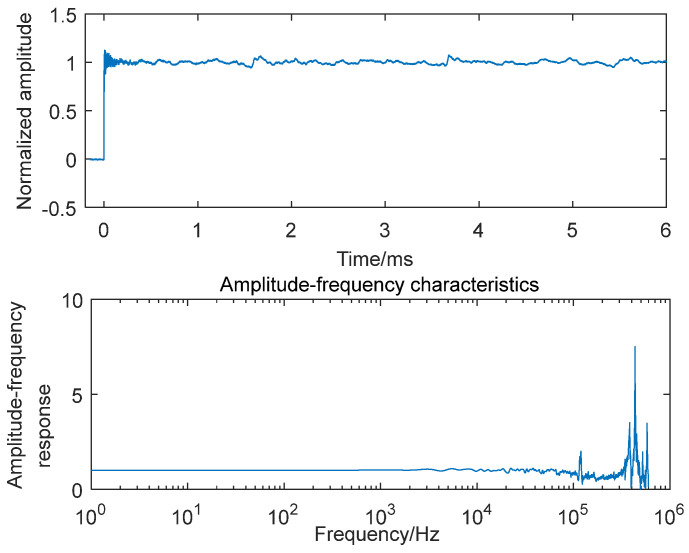
Step response and amplitude−frequency characteristics of the sensor.

**Figure 6:**
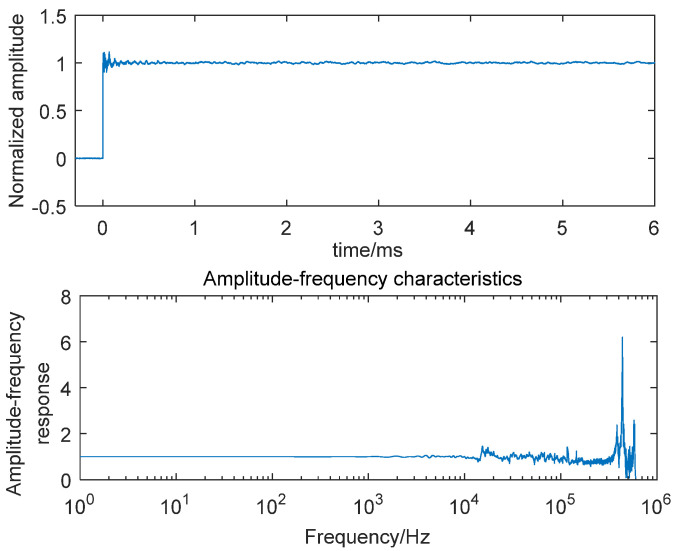
Step response and amplitude−frequency characteristics of the sensor with an additional mechanical structure.

**Figure 7:**
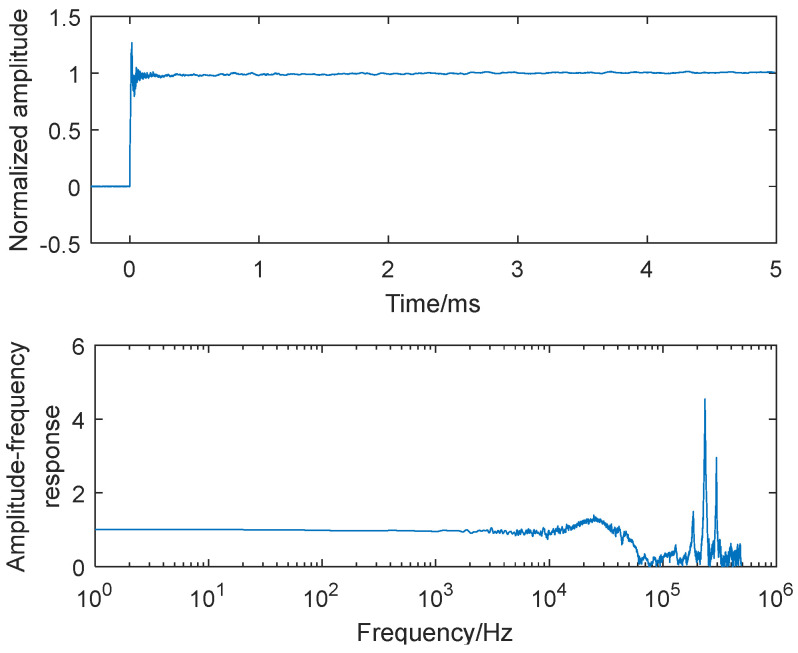
Step response and amplitude−frequency characteristics of the sensor with an additional mechanical structure and a 0.3 mm thickness of silicone grease.

The experimental results in Figure 5, Figure 6, and Figure 7 present the step responses and frequency characteristics across the three configurations. Figure 5 shows two resonant peaks at approximately 120 kHz (originating from the sensor’s mounting thread) and 430 kHz (corresponding to the sensor’s natural frequency). In Figure 6, with the addition of mechanical structures, three distinct resonant frequencies emerge: 15 kHz (mechanical structure’s mounting thread), 120 kHz (sensor’s mounting thread), and 430 kHz (sensor’s natural frequency). In Figure 7, the complete assembly with silicone grease exhibits modified peaks at 24 kHz and 230 kHz, representing combined effects from the sensor, mechanical structure, and thermal insulation layer. These findings confirm that both the mechanical buffer and 0.3 mm silicone grease substantially modify the system’s dynamic behavior, particularly through natural frequency reduction and bandwidth narrowing, thus necessitating dynamic recalibration whenever boundary conditions or mechanical attachments are altered in the enhanced shock wave pressure sensor system.

### 2.4. Dynamic Characteristics of Enhanced Shock Wave Pressure Sensor System

**Figure 8:**
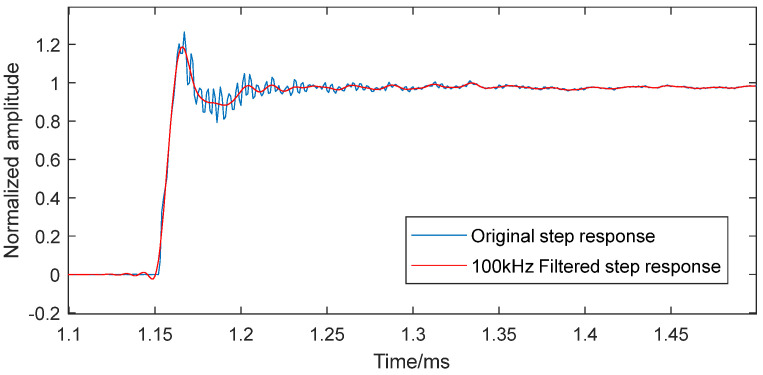
The step response in the dynamic calibration test.

Prior to analysis, the step response data underwent comprehensive preprocessing: zero drift and platform trends were eliminated via polynomial fitting, followed by signal normalization to extract the sensor system’s normalized dynamic characteristics, with the final application of a 100 kHz low-pass filter to prevent aliasing and suppress high-frequency noise. As shown in Figure 8, the buffered PCB113B sensor demonstrates an 18% overshoot and 45 μs response time in its step response.

**Figure 9:**
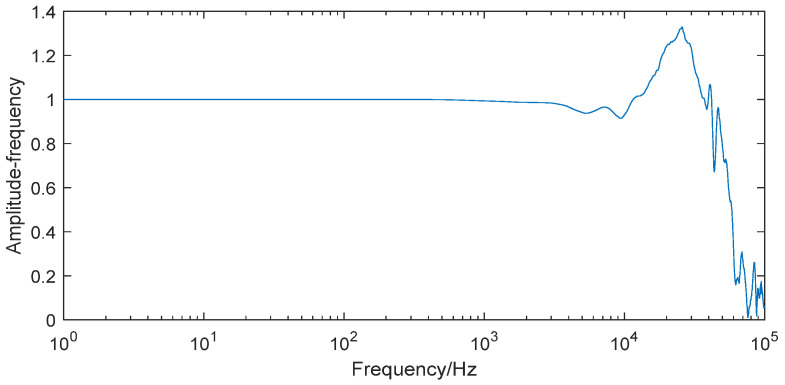
The dynamic characteristics of the enhanced pressure sensor system.

The dynamic characteristics of the enhanced PCB113B shock wave pressure sensor system were determined through shock tube calibration using differential methods. In Figure 9, the dynamic characteristics of the enhanced PCB113B pressure sensor system reveal three critical limitations: an upper bandwidth limit below 10 kHz, insufficient for covering the measured signal’s effective bandwidth; pronounced dynamic distortion across the 100 kHz frequency range; and the consequent introduction of significant measurement errors. These findings demonstrate the necessity of implementing advanced modeling and compensation techniques to restore measurement accuracy and minimize error propagation in shock wave applications.

## 3. Modeling and Compensation Methodology Based on BP Neural Network

### 3.1. BP Neural Network

**Figure 10:**
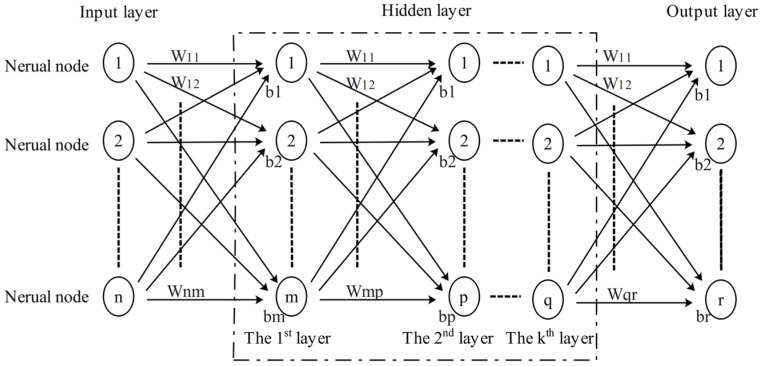
The composition of a BP neural network.

The backpropagation (BP) neural network is a multilayer feedforward network with an input layer, multiple hidden layers, and an output layer, consisting of interconnected neural nodes [20], as shown in Figure 10. Its training involves bidirectional information flow: forward propagation transforms input signals through weighted connections and activations across layers and backward propagation calculates output errors and distributes them backward using calculus, enabling weight updates via gradient descent.

During forward propagation, input data propagate from the input layer through hidden layers to the output layer, where connection weights and output values are generated for subsequent steepest descent optimization.

The input *S_i_* of the *i*-th neural node of the hidden layer is calculated as shown in Formula (1).

(1) $$S_{i}=\sum_{j=1}^{N}w_{ji}x_{j}-\theta_{i}$$

The output *O_i_* of the *i*-th neural node in the hidden layer is obtained as shown in Formula (2).

(2) $$O_{i}=\Phi (S_{i})$$

The input *S_k_* of the *k*-th neural node of the output layer is derived as shown in Formula (3).

(3) $$S_{k}=\sum_{i=1}^{Q}w_{ik}O_{i}-\phi_{k}$$

The output *O_k_* of the *k*-th neural node of the output layer is shown in Formula (4).

(4) $$O_{k}=\Psi (S_{k})$$

where *w_ji_* is the connection weight from the *j*-th neural node of the input layer to the *i*-th neural node of the hidden layer, *x_j_* is the input signal of the *j*-th neural node of the input layer, *θ_i_* is the threshold of the *i*-th neural node in the hidden layer, *w_ik_* is the connection weight from the *i*-th neural node of the hidden layer to the *k*-th neural node of the output layer, *φ_k_* is the threshold of the *k*-th neural node in the output layer, Φ is the excitation function of the neural node in the hidden layer, Ψ is the excitation function of the neural node in the output layer, and the Sigmoid function is often used as the excitation function.

If the output fails to converge, backward propagation using the Widrow–Hoff learning rule should be performed to iteratively adjust the hidden and output layer connection weights.

Assuming that P is the quantity of training samples and M is the quantity of output nodes, the overall error, EP, is defined as shown in Formula (5).

(5) $$E_{P}=\frac{1}{2}\sum_{p=1}^{P}\sum_{k=1}^{M}(y_{k}^{p}-O_{k}^{p}{)}^{2}$$

where *y_k_* represents the expected output values of the neural network and *O_k_* represents the actual output values of the neural network. Based on the overall error criterion, first, the input weight *w_ik_* of the output layer and the threshold *φ_k_* in the output layer should be adjusted in turn through the gradient searching strategy. Then the input weight *w_ji_* of the hidden layer and the threshold *θ_i_* in the hidden layer should be adjusted.

The input weight correction formula of the hidden layer is shown in Formula (6).

(6) $$w_{ji}(n+1)=\Delta w_{ji}(n)+w_{ji}(n)$$

The threshold correction formula of the hidden layer is present in Formula (7).

(7) $$\theta_{i}(n+1)=\Delta \theta_{i}(n)+\theta_{i}(n)$$

The input weight correction formula of the output layer is illustrated in Formula (8).

(8) $$w_{ik}(n+1)=\Delta w_{ik}(n)+w_{ik}(n)$$

The threshold correction formula of the output layer is shown in Formula (9).

(9) $$\varphi_{k}(n+1)=\Delta \varphi_{k}(n)+\varphi_{k}(n)$$

where Δ*w_ji_*, Δ*θ_i_*, Δ*w_ik_*, and Δ*φ_k_* can be derived by calculating the partial derivatives of *w_ji_*, *θ_i_*, *w_ik_*, and *φ_k_* with respect to the overall error *E_P_*, respectively.

The forward and backward propagations alternate iteratively until the neural network’s outputs meet the target specifications.

### 3.2. Modeling of Pressure Sensor System Based on Neural Network

Neural network modeling typically employs two architectures: the forward parallel model and the forward series–parallel model. The forward parallel model utilizes the network’s previous outputs as subsequent inputs to enhance nonlinear approximation capability, though this imposes stringent requirements on the initial coefficients and improper initialization may compromise output accuracy or prevent convergence. When the initial coefficients are uncertain, the forward series–parallel model is preferred, with the pressure sensor’s nonlinear series–parallel model being constructed using the Nonlinear Autoregressive Moving Average (NARMA) [28] framework, as expressed in Equation (10).

(10) y[k]=f(y[k−1],…,y[k−n],…,x[k],…,x[k−n])

where $y\left[k\right]$ is the current output value,$y\left[k-1\right],\ldots ,y\left[k-n\right]$ are past output values, $x\left[k\right]$ is the current input value, $x\left[k-1\right],\ldots ,x[k-n]$ are past input values, and n is the order.

The pressure sensor system’s neural network model is established through the following methodology: using an ideal step signal as the input and the sensor system’s step response as the output, the input layer incorporates current/past input values and past output values, while the output layer represents current output values. This BP neural network architecture requires careful selection of key parameters including model order for optimal performance.

### 3.3. Dynamic Compensation of the Pressure Sensor System Based on a Neural Network

When the sensor system fails to meet undistorted measurement requirements, traditional dynamic compensation employs a digital filter design, which inherently assumes linear time-invariant system behavior, thereby introducing nonlinear errors that degrade compensation performance. To address this limitation, this study develops a forward series–parallel dynamic compensation model using a BP neural network, leveraging their superior nonlinear characterization capabilities. Following the NARMA framework (analogous to the pressure sensor system’s neural network modeling approach), the proposed compensation model is mathematically expressed in Equation (11).

(11) x’[k]=g(x’[k−1],…,x’[k−n],…,y’[k],…y’[k−n])

where $x’\left[k\right]$ is the current output value, $x’\left[k-1\right],\ldots ,x’\left[k-n\right]$ are past output values, $y’\left[k\right]$ is the current input value, $y’\left[k-1\right],\ldots ,y’[k-n]$ are past input values, and n is the order.

To effectively train the compensation model’s BP neural network weights, we configured the input values as the step response signal generated when the ideal step signal processes data through the pre-established neural network model rather than using raw dynamic calibration test data, while designating the ideal step signal itself as the target output values. The input layer integrates current inputs, historical inputs, and past outputs, with the output layer representing current predicted values, thereby constructing a complete BP neural network-based dynamic compensation model for the pressure sensor system.

## 4. Modeling and Compensation Practice of Enhanced Pressure Sensor System

### 4.1. Neural Network Model of Enhanced Pressure Sensor System

Based on the dynamic calibration test, we first established a neural network model for the enhanced pressure sensor system. According to the Universal Approximation Theorem, a BP neural network with one hidden layer can approximate any continuous nonlinear function to any desired precision, provided that the number of neurons in the hidden layer is sufficient. Additionally, a single hidden layer offers greater advantages in terms of training efficiency and generalization ability. Therefore, the neural network model was configured with one hidden layer. The maximum epoch was set to 100 steps, with the target error approaching zero. The learning rate was set to 0.1, the Sigmoid function was used as the activation function, and the Levenberg–Marquardt training function was selected. To determine the model order and the number of neural nodes, we followed this principle: when increasing or decreasing the parameters further no longer significantly alters the residual sum of squares, the current values are deemed optimal.

First, we set the quantity of neural nodes to 5. To determine the optimal model order, we tested values ranging from 2 to 9. As shown in Table 1, the computational time exhibits an upward trend while the residual sum of squares (RSS) decreases as the model order increases. Notably, when the order reaches 7, the reduction in RSS becomes marginal. Therefore, the optimal model order is determined to be 7.

After setting the model order to 7, we need to determine the optimal number of neural nodes. Table 2 lists the typical computing time and the residual sum of squares when the node count ranges from 2 to 9.

**Figure 11:**
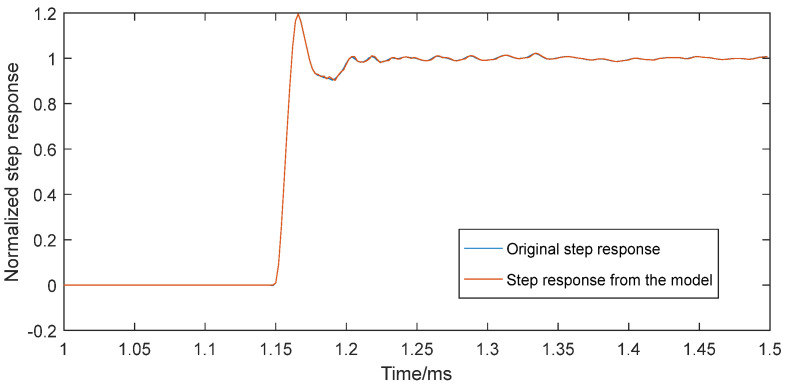
The original step response and the step response from the neural network model.

**Figure 12:**
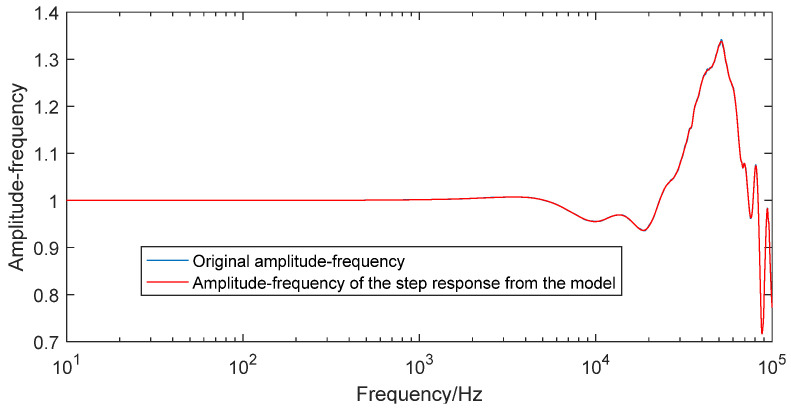
The dynamic characteristics from the calibration test and the neural network model.

As shown in Table 2, as the number of nodes increases, computing time rises, while the residual sum of squares gradually decreases. Once the node count reaches 7, the residual sum of squares exhibits only a marginal further reduction. Consequently, 7 neural nodes were selected for the final model, with both the model order and neural node count set to 7 to improve computational efficiency and simplify the model. This configuration yields an accurate input–output neural network model for the enhanced pressure sensor system. Feeding an ideal step signal into the network produced its step response. Figure 11 compares this simulated response with the experimentally measured step response from dynamic calibration; Figure 12 juxtaposes the corresponding dynamic characteristics derived from the two curves. The close agreement between both pairs of curves confirms the high fidelity and reliability of the neural network model.

### 4.2. Dynamic Compensation Model of Enhanced Pressure Sensor System

Using the step response signal shown in Figure 11 generated by the neural network model, we can now build a dynamic compensation network. In this compensation model, the simulated step response serves as the input, while the near-ideal step signal is taken as the desired output. A single hidden layer is adopted, training is run for 100 iterations, and convergence is declared when the model error approaches zero. Following the selection principle outlined in Section 4.1, the model order is set to 5 and the number of hidden nodes to 5, yielding the final dynamic compensation model for the pressure sensor.

**Figure 13:**
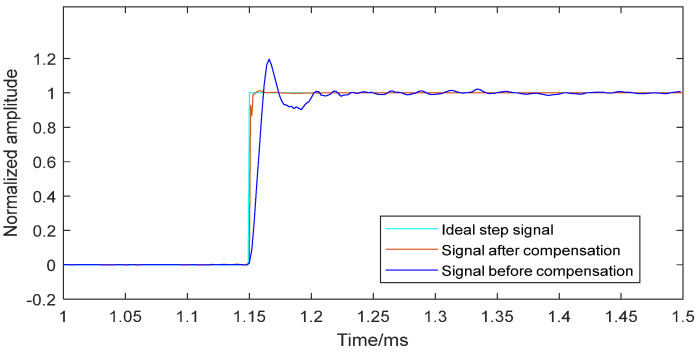
The original step and the step signal from the neural network model.

Figure 13 compares the ideal step signal, the signal before compensation, and the signal reconstructed by the neural network dynamic compensation model. The near-perfect overlap of the ideal step signal and the signal after compensation confirms the effectiveness of the compensation model for the sensor system.

### 4.3. Reliability Verification of Dynamic Compensation Model

**Figure 14:**
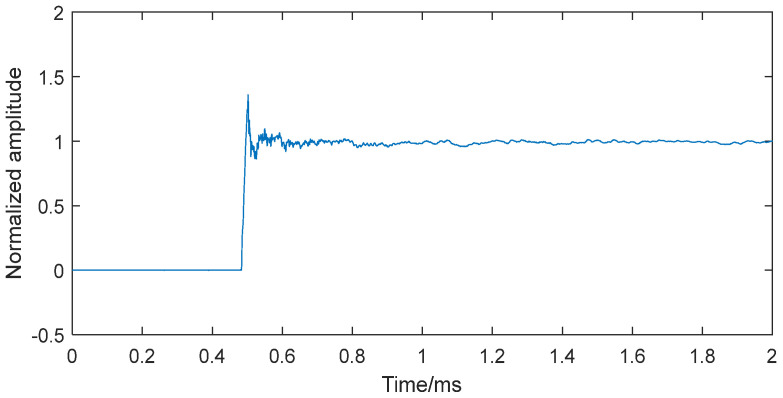
The new normalized step response from another dynamic calibration test.

**Figure 15:**
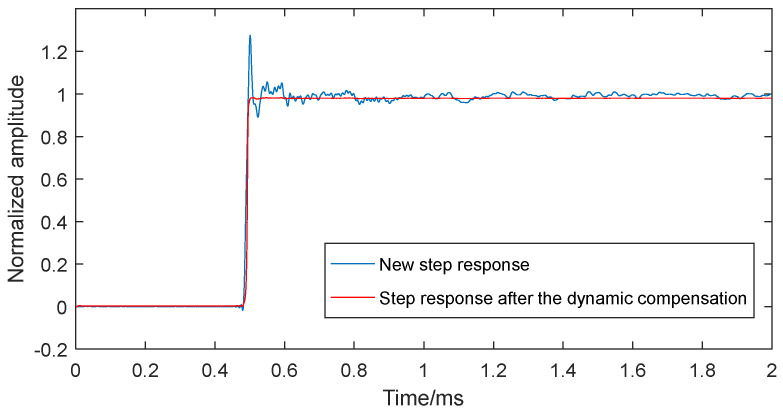
The 100 kHz filtered new step response and the step signal from the dynamic compensation model.

To validate the reliability of the established dynamic compensation model, a new normalized step response was obtained from an independent dynamic calibration test, as shown in Figure 14. This signal differs in platform length and sampling frequency from that shown in Figure 8. After processing it through the neural network compensation model, the compensated signal, shown in Figure 15, closely matched the ideal step input, confirming the compensation model’s effectiveness. The results demonstrate that the BP neural network-based compensation reliably enhances the sensor system’s dynamic performance.

### 4.4. Dynamic Compensation of Measured Blast Shock Wave Pressure Signal in Explosion Test

**Figure 16:**
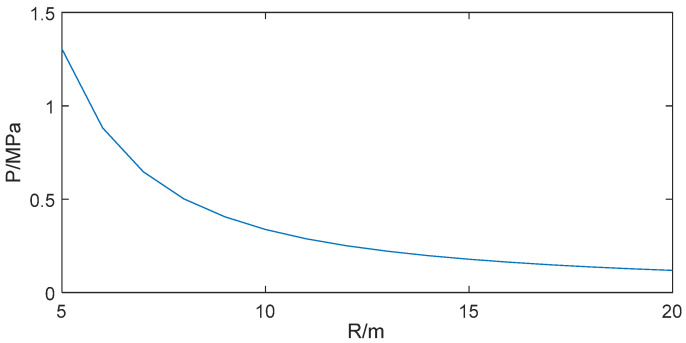
The relationship between reflected peak overpressure and distance.

In the explosion test, the reflected blast shock wave pressure was measured with the enhanced PCB113B sensor system. According to blast mechanics, the reflected peak overpressure of the blast shock wave can be calculated as follows [29]:

(12) $$\Delta P_{1}=0.84\frac{\sqrt[{3}]{W}}{R}+2.7{\left(\frac{\sqrt[{3}]{W}}{R}\right)}^{2}+7{\left(\frac{\sqrt[{3}]{W}}{R}\right)}^{3}$$

(13) $$\Delta P_{2}=2\Delta P_{1}+\frac{6\Delta P_{1}^{2}}{\Delta P_{1}+7P_{0}}$$

where Δ*P*_1_ is the shock wave incident peak overpressure, Δ*P*_2_ is the reflected peak overpressure, *W* is the equivalent weight of TNT, which is 1 kg, *R* is the distance from the blast center to the measurement point, and *P*_0_ is the atmospheric pressure, which is 0.1 MPa. The variation trend of the reflected peak overpressure with the change in distance R is shown in Figure 16.

**Figure 17:**
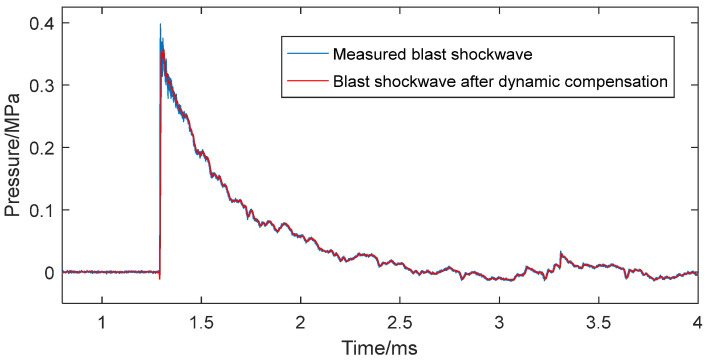
Blast shock wave pressure signal before and after dynamic compensation.

For a 1 kg TNT charge at a 10 m standoff, blast mechanics calculations yield an incident peak overpressure of 0.12 MPa and a reflected peak overpressure of 0.34 MPa. Figure 17 compares the output before and after dynamic compensation for the compression phase of the blast shock wave, showing the reflected peak overpressure dropping from 0.40 MPa (uncompensated) to 0.35 MPa (compensated).

Relative to the theoretical peak overpressure of 0.34 MPa, the uncompensated signal showed an 18% error, which was mainly caused by the performance of instruments, the parasitic effects (even if they were suppressed) in the explosion environment, the installation of the sensor, and the nonlinearity in the dynamic characteristics of the sensor system [30]. In comparison, the dynamically compensated signal reduces this error to 3%. Dynamic modeling and compensation based on the BP neural network can effectively reduce the uncertainty induced by the nonlinearity in the dynamic characteristics of the sensor system. The above results demonstrate that neural network-based modeling and compensation significantly and effectively correct blast shock wave pressure signals and ensure measurement accuracy.

## 5. Conclusions

The accurate measurement of blast shock wave pressure during its compression phase is critical for assessing explosive destructive power, yet it faces significant challenges due to the complex blast environment characterized by steep rising edges and short signal durations, compounded by parasitic effects like mechanical vibrations and thermal shocks. This study addressed these challenges by developing an enhanced sensor system integrating a specialized buffer device to mitigate disturbances, followed by the BP neural network-based modeling and compensation method to restore the measurement accuracy of compression-phase pressure data.

The research demonstrated that while the buffer device effectively reduced mechanical and thermal interference, it introduced dynamic performance limitations, notably narrowing the sensor system’s operational bandwidth below the required 10 kHz range for undistorted blast wave measurements. Through dynamic calibration using a double-diaphragm shock tube, the study systematically characterized these modified dynamic properties, revealing distinct resonant frequencies at 15 kHz, 120 kHz, and 430 kHz for the mechanically enhanced system, which further shifted to 24 kHz and 230 kHz with the addition of silicone grease thermal insulation.

The core innovation of this work lies in its application of BP neural networks to overcome the inherent nonlinearities that traditional linear time-invariant system modeling methods fail to address. By establishing a forward series–parallel model based on the NARMA framework and optimizing key parameters through rigorous testing, the neural network model achieved exceptional fidelity with residual sums of squares as low as 5.7 × 10^−4^. The dynamic compensation model successfully reconstructed near-ideal step responses from distorted signals, with experimental validation showing near-perfect overlap between compensated and theoretical signals. Practical implementation in explosion tests with 1 kg TNT charges demonstrated the method’s effectiveness, reducing peak overpressure measurement errors from 18% to just 3% compared to theoretical calculations. These results validate the BP neural network’s superior capability in handling the sensor system’s nonlinear dynamics compared to conventional frequency-domain compensation methods.

In this study, three key contributions emerged, all centered on improving compression-phase measurement. First, it provides a comprehensive solution combining mechanical design with intelligent algorithms to address the unique challenges of compression-phase pressure measurement. Second, it establishes a robust methodology for neural network-based sensor modeling and compensation. Third, it offers practical validation in real explosive environments. The successful integration of hardware enhancements and software compensation addresses a critical gap in accurate dynamic pressure measurement, particularly for applications requiring both vibration resistance and a wide bandwidth. This research has significant implications for explosive testing, structural safety assessment, and sensor development, paving the way for more reliable data acquisition in high-energy environments.

## Figures and Tables

**Table 1:** Typical computing time and the residual square sum for different model orders.

The Quantity of Neural Nodes	The Model Order	The Computing Time	The Residual Square Sum
5	2	0.3037	14 × 10^−4^
	3	0.3330	12 × 10^−4^
	4	0.3057	9.8 × 10^−4^
	5	0.3517	9.0 × 10^−4^
	6	0.3423	7.8 × 10^−4^
	7	0.3635	6.1 × 10^−4^
	8	0.3530	6.3 × 10^−4^
	9	0.3694	6.7 × 10^−4^

**Table 2:** Typical computing time and the residual square sum for different quantities of neural nodes.

The Model Order	The Quantity of Neural Nodes	The Computing Time	The Residual Square Sum
7	2	0.2624	8.9 × 10^−4^
	3	0.2861	7.2 × 10^−4^
	4	0.3057	6.7 × 10^−4^
	5	0.3517	6.6 × 10^−4^
	6	0.3423	6.1 × 10^−4^
	7	0.3635	5.7 × 10^−4^
	8	0.3530	5.6 × 10^−4^
	9	0.3694	5.5 × 10^−4^

## Data Availability

The raw data supporting the conclusions of this article will be made available by the authors on request.

## References

[1] Godio M., Portal N.W., Flansbjer M., Magnusson J., Byggnevi M. (2021). Experimental and numerical approaches to investigate the out-of-plane response of unreinforced masonry walls subjected to free far-field blasts. Eng. Struct..

[2] Medda A., Funk R., Ahuja K., Kamimori G. (2021). Measurements of Infrasound Signatures from Grenade Blast During Training. Mil. Med..

[3] Wang J., Cao J., Yao C., Wu Y. (2018). Force sensor model identification and dynamic compensator design. Des. Res..

[4] Wang H., Zeng Q., Zhang Z., Wang H. (2022). Research on Temperature Compensation of Multi-Channel Pressure Scanner Based on an Improved Cuckoo Search Optimizing a BP Neural Network. Micromachines.

[5] Svete A., Kutin J. (2022). Identifying the high-frequency response of a piezoelectric pressure measurement system using a shock tube primary method. Mech. Syst. Signal Process..

[6] Yang F., Kong D., Wang F., Kong L. (2019). A traceable dynamic calibration research of the measurement system based on quasi-static and dynamic calibration for accurate blast. Meas. Sci. Technol..

[7] Gu T., Shang F., Kong D., Xu C. (2019). Absolute quasi-static calibration method of piezoelectric high-pressure sensor based on force sensor. Rev. Sci. Instrum..

[8] Pallas N.-P., Kellaris K., Bouris D. (2024). Dynamic calibration of complex tubing systems using a single pressure measurement device. J. Wind Eng. Ind. Aerodyn..

[9] Pereira J.D. (2024). Pressure Sensors: Working Principles of Static and Dynamic Calibration. Sensors.

[10] Yao Z., Li Y., Ding Y., Wang C., Yao L., Song J. (2022). Improved shock tube method for dynamic calibration of the sensitivity characteristic of piezoresistive pressure sensors. Measurement.

[11] Svete A., Amer E., Jönsson G., Kutin J., Arrhén F. (2023). A method for correcting the high-frequency mechanical vibration effects in the dynamic calibration of pressure measurement systems using a shock tube. Mech. Syst. Signal Process..

[12] Seyedpour S.M., Lambers L., Rezazadeh G., Ricken T. (2023). Mathematical modelling of the dynamic response of an implantable enhanced capacitive glaucoma pressure sensor. Meas. Sens..

[13] Nadezhdin I.S., Goryunov A.G., Svinolupov Y.G., Zadorozhnaya O.J. (2019). Study of the metrological characteristics of the hydrostatic pressure sensor. Sens. Rev..

[14] Huang X., Zhang X. (2020). Investigating the advanced characteristics of SiC based piezoresistive pressure sensors. Mater. Today Commun..

[15] Xue L., Du H., Pei D., He Z., Cao X. (2014). Design of Shock Wave Overpressure Measurement System Based on ICP Senso. Instrum. Tech. Sens..

[16] Pieniazek J., Ciecinski P. (2020). Temperature and Nonlinearity Compensation of Pressure Sensor With Common Sensors Response. IEEE Trans. Instrum. Meas..

[17] Xu B., Han T., Liu H., Wang X., Ju M. (2020). Dynamic Compensation of Piezoresistive Pressure Sensor Based on Sparse Domain. J. Sens..

[18] Liu M., Wang Z., Jiang P., Yan G. (2024). Temperature Compensation Method for Piezoresistive Pressure Sensors Based on Gated Recurrent Unit. Sensors.

[19] Wang H., Li J. (2022). Machine Learning and Swarm Optimization Algorithm in Temperature Compensation of Pressure Sensors. Sensors.

[20] Kang M.A., Park C.H. (2023). Prediction of Peak Pressure by Blast Wave Propagation Between Buildings Using a Conditional 3D Convolutional Neural Networkr. IEEE Access.

[21] Zhu F., Liang Q. (2021). Rethink of Orthographic Constraints on RNN and Its Application in Acoustic Sensor Data Modeling. IEEE Internet Things J..

[22] Pierre A.A., Akim S.A., Semenyo A.K., Babiga B. (2023). Peak Electrical Energy Consumption Prediction by ARIMA, LSTM, GRU, ARIMA-LSTM and ARIMA-GRU Approaches. Energies.

[23] Zhang K., Zhu J., He M., Jiang Y., Zhu C., Li D., Kang L., Sun J., Chen Z., Wang X. (2022). Research on Intelligent Comprehensive Evaluation of Coal Seam Impact Risk Based on BP Neural Network Model. Energies.

[24] Vitolo P., Liguori R., Di Benedetto L., Rubino A., Pau D., Licciardo G.D. (2025). Real-time neural network-based thermal stress compensation for pressure sensors in precision localization systems. Microprocess. Microsyst..

[25] Xu W., Feng X., Xing H. (2019). Modeling and Analysis of Adaptive Temperature Compensation for Humidity Sensors. Electronics.

[26] Cheng L., Huang F., Wu H., Dong H., Tian S. (2024). Comparative analysis of blast load transfer and structural damage in multi-cabin structures during air and underwater explosions. Ocean Eng..

[27] Diao K., Yao Z., Wang Z., Liu X., Wang C., Shang Z. (2019). Investigation of vibration effect on dynamic calibration of pressure sensors based on shock tube system. Measurement.

[28] Bulucu P., Soydemir M.U., Şahin S., Kocaoğlu A., Güzeliş C. (2020). Learning Stable Robust Adaptive NARMA Controller for UAV and Its Application to Twin Rotor MIMO Systems. Neural Process. Lett..

[29] Chen H., Tao G., Pu Y. (2010). The Measurement of Overpressure of Shock Wave and Analysis of TNT Equivalent. Initiat. Pyrotech..

[30] Gu T., Kong D., Shang F., Chen J. (2018). A calculation and uncertainty evaluation method for the effective area of a piston rod used in quasi-static pressure calibration. Meas. Sci. Technol..

